# Reduction of pulmonary toxicity of metal oxide nanoparticles by phosphonate-based surface passivation

**DOI:** 10.1186/s12989-017-0193-5

**Published:** 2017-04-21

**Authors:** Xiaoming Cai, Anson Lee, Zhaoxia Ji, Cynthia Huang, Chong Hyun Chang, Xiang Wang, Yu-Pei Liao, Tian Xia, Ruibin Li

**Affiliations:** 1 Center for Genetic Epidemiology and Genomics, School of Public Health, Jiangsu Key Laboratory of Preventive and Translational Medicine for Geriatric Diseases, Medical College of Soochow University, Suzhou, 215123 China; 2 School for Radiological and Interdisciplinary Sciences (RAD-X), Collaborative Innovation Center of Radiation Medicine of Jiangsu Higher Education Institutions, Jiangsu Provincial Key Laboratory of Radiation Medicine and Protection, Soochow University, Suzhou, 215123 China; 3 Department of Medicine, University of California, Los Angeles, California 90095 USA; 4 California NanoSystems Institute, University of California, Los Angeles, California 90095 USA; 5 Department of Chemical and Biomolecular Engineering, University of California, Los Angeles, California 90095 USA; 6 Department of Microbiology, Immunology, and Molecular Genetics, University of California, Los Angeles, California 90095 USA

**Keywords:** Metal oxides, Nanotoxicity, Functionalization, Lung toxicity, Surface coating, Inhalation, Inflammation

## Abstract

**Background:**

The wide application of engineered nanoparticles has induced increasing exposure to humans and environment, which led to substantial concerns on their biosafety. Some metal oxides (MOx) have shown severe toxicity in cells and animals, thus safe designs of MOx with reduced hazard potential are desired. Currently, there is a lack of a simple yet effective safe design approach for the toxic MOx. In this study, we determined the key physicochemical properties of MOx that lead to cytotoxicity and explored a safe design approach for toxic MOx by modifying their hazard properties.

**Results:**

THP-1 and BEAS-2B cells were exposed to 0–200 μg/mL MOx for 24 h, we found some toxic MOx including CoO, CuO, Ni_2_O_3_ and Co_3_O_4_, could induce reactive oxygen species (ROS) generation and cell death due to the toxic ion shedding and/or oxidative stress generation from the active surface of MOx internalized into lysosomes. We thus hypothesized that surface passivation could reduce or eliminate the toxicity of MOx. We experimented with a series of surface coating molecules and discovered that ethylenediamine tetra (methylene phosphonic acid) (EDTMP) could form stable hexadentate coordination with MOx. The coating layer can effectively reduce the surface activity of MOx with 85-99% decrease of oxidative potential, and 65-98% decrease of ion shedding. The EDTMP coated MOx show negligible ROS generation and cell death in THP-1 and BEAS-2B cells. The protective effect of EDTMP coating was further validated in mouse lungs exposed to 2 mg/kg MOx by oropharyngeal aspiration. After 40 h exposure, EDTMP coated MOx show significant decreases of neutrophil counts, lactate dehydrogenase (LDH) release, MCP-1, LIX and IL-6 in bronchoalveolar lavage fluid (BALF), compared to uncoated particles. The haematoxylin and eosin (H&E) staining results of lung tissue also show EDTMP coating could significantly reduce the pulmonary inflammation of MOx.

**Conclusions:**

The surface reactivity of MOx including ion shedding and oxidative potential is the dominated physicochemical property that is responsible for the cytotoxicity induced by MOx. EDTMP coating could passivate the surface of MOx, reduce their cytotoxicity and pulmonary hazard effects. This coating would be an effective safe design approach for a broad spectrum of toxic MOx, which will facilitate the safe use of MOx in commercial nanoproducts.

**Electronic supplementary material:**

The online version of this article (doi:10.1186/s12989-017-0193-5) contains supplementary material, which is available to authorized users.

## Background

Metal oxides (MOx) are the most produced nanomaterials because of their unique physicochemical properties including catalytic activity, antibacterial capability, chemical stability, electrical, thermal and mechanical characteristics. MOx are now widely used in electronics, mechanics, biomedicine, catalysis, cosmetics, painting, polishing, *etc*. [1–3]. The increasing production as well as wide use of MOx has led to substantial concerns on their potential hazardous effects to both humans and environment [4]. Some MOx have been reported to induce reactive oxygen species (ROS) generation [5], mitochondrial damage [6], cytochrome C release [7], DNA damage [8], inflammatory cytokine release [9–11] and cell death[12]. After exposure to animal lungs, certain MOx can trigger acute pulmonary inflammation [13]. In addition, other hazardous effects including autophagy interferences and pro-fibrogenic effects were also reported [11, 14]. The toxicity induced by MOx has been demonstrated to closely correlate with their surface properties such as electronic property (band gap), functional groups, dissolution, *etc*. [10, 13, 15]. Therefore, we hypothesized that surface passivation could be an effective approach to engineer safe MOx.

Currently, safe design approaches including surface coating [16, 17], core-shell structure [18, 19], doping [20] and geometric control [21] have been developed to reduce the hazard effects of nanomaterials. Among these safe design approaches, surface coating is a simple and effective post-modification method, which can be extended to a variety of nanoparticles with similar surface properties. For example, Wang et al. found that pluronic molecules can effectively reduce the hazard potential of carbon nanotubes [22], and this coating approach could be extended to protect other carbonaceous nanomaterials including graphene and graphene oxide [23] or other 2-D materials such as MoS_2_ [24]. Since MOx induced toxicity are mainly based on surface reactivity or ion shedding from MOx surface, surface coating would have the opportunity to reduce both. However, to date, there is lack of an effective surface coating approach for a broad-spectrum of MOx.

In this communication, we experimented with a series of coating molecules on selected MOx to discover the most effective for surface passivation. The desired coating molecule could reduce the surface reactivity and/or dissolution of MOx, and thus reduce the hazard potential of MOx *in vitro* and *in vivo*.

## Methods

### Materials

CoO and Ni_2_O_3_ nanoparticles were purchased from SkySpring Nanomaterials (Houston, TX); CuO, Co_3_O_4_ and TiO_2_ nanoparticles were synthesized by a flame spray pyrolysis reactor; Ethylenediamine tetra(methylenephosphonic acid) (EDTMP) was purchased from Tokyo Chemical Industry Co. (Chuo-ku, Tokyo, Japan); poly(vinylpyrrolidone) (PVP) and citrate were purchased from Sigma-Aldrich (St. Louis, MO). ELISA kits for detection of IL-6 and MCP-1, purchased from BD Biosciences (San Jose, CA). LIX ELISA kit was purchased from R&D Systems (Minneapolis, MN). Hoechst 33342 were purchased from Life Technologies (Grand Island, NY, USA). The lactate dehydrogenase (LDH) and MTS assay kits were obtained from Promega (Madison, WI) ATP assay kit was purchased from Perkin-Elmer (Boston, MA).

### Nanoparticle Characterization

All of the nanoparticles were provided in powder form. Transmission electron microscopy (TEM, JEOL 1200 EX instrument) was used to observe the shapes and primary sizes of the nanoparticles. Samples were prepared by placing a drop of the aqueous nanoparticle suspension on a carbon-coated TEM grid and waiting until the water evaporates. To determine the primary particle size, 100 nanoparticles were counted in the TEM images of each MOx. Dynamic light scattering (DLS, Dynapro Plate Reader, Wyatt Technology) was performed to determine the particle size in water and the cell culture media. Zeta-potential measurement of the nanoparticle suspensions was performed using a ZetaPALS instrument (Zeta Potential Analyzer, Brookhaven Instruments Corporation, Holtsville, NY). Metal dissolution was determined by inductively coupled plasma optical emission spectrometry (ICP-OES) (ICPE-9000, Shimadzu, Japan). Fourier transform infrared (FTIR) spectra were collected using a Bruker Vertex 70 instrument.

### Confocal Microscopy Imaging

For cellular biodistribution study, THP-1 cells were incubated with 25 μg/mL FITC labeled TiO_2_, Co_3_O_4_ and CuO nanoparticle suspensions for 16 h. For intracellular ROS visualization, MitoSOX™ reagent (Invitrogen, USA) was diluted in cell culture media at 5 μM and applied to cells pretreated with 100 μg/mL coated or uncoated MOx for 16 h, followed by 10 min incubation at 37 °C. Then the cells were collected and washed with PBS for 3 times. After fixing in 4% paraformaldehyde, cells were stained with Hoechst 33342 or Alexa Fluor 594 labeled anti-LAMP1 to visualize the nuclei and lysosomes, respectively, by Leica confocal SP2 1P/FCS microscope.

### Surface coating

We compared the coating efficiency of coating molecules (citrate, PVP, EDTMP, Aminomethyl phosphonic acid, Polyethylene glycol, 3-bromopropyl phosphonic acid, *etc*.) at a 0.5 ~ 5 weight ratio of coating agent *vs* MOx, and found that a weight ratio of 2 was sufficient for effective surface passivation. Therefore, a 2 mg amount of MOx was dispersed in 10 mL of DI H_2_O containing coating molecules at concentration of 400 μg/mL. MOx were reacted with these coating molecules for 24 h at room temperature with magnetic stirring. The particle solutions were centrifuged at 135, 558 g/min for 1 h to collect the particle pellets. After washing with DI H_2_O, the coated MOx were stored at 4 °C for further characterization and use.

### Preparation of MO nanoparticle suspensions in media

Coated and uncoated metal oxide nanoparticles were suspended in DI water at 5 mg/mL as stock solutions. These suspensions were sonicated at 100 W output with frequency of 42 kHz for 15 min in a water sonicator (Branson, Danbury, CT, USA, model 2510). The suspensions were used as stock solutions for further dispersion in cell culture media or PBS. An appropriate amount of each stock solution was added to cell culture media or PBS to achieve the desired final concentration. For better dispersion, BSA was added to BEGM medium or PBS at 0.2 mg/mL, before the addition of nanoparticles. The diluted MO suspensions were dispersed using a sonication probe (Sonics & Materials, USA) at 32 W for 10 s at the desired final concentration before further use.

### Assessment of the oxidative capability of MOx

The oxidative capacity of MOx were determined by a method as described before [15]. 2’,7’-dichlorodihydro-fluorescein diacetate (H_2_DCFDA) was used to evaluate the oxidation capacity of MOx. The 2',7'-dichlorofluorescein (DCF) working solution was prepared by mixing 50 μg of H_2_DCFDA with 280 μL 0.01 M NaOH. The resulting solution was incubated for 30 min, and diluted with 1720 μL of a sodium phosphate buffer (25 mmol/L, pH = 7.4) to form 25 μg/mL DCF solution. 96 μL aliquots of 25 μg/mL DCF working solutions were added into each well of a 96 multiwell black plate (Costar, Corning, NY). A 4 μL amount of 5 mg/mL nanoparticle suspension was subsequently added to each well, followed by 2 h incubation. DCF fluorescence emission spectra in the range of 500–600 nm were collected using a SpectraMax M5 microplate reader with an excitation wavelength of 490 nm.

### Cell viability test

BEAS-2B and THP-1 cells were obtained from ATCC (Manassas, VA, USA). BEAS-2B cells were suspended in BEGM medium at a density of 1 × 10^5^/mL, and then added in 96-well plates with 100 μL/well. Aliquots of 3 × 10^4^ THP-1 cells were seeded in 0.1 mL RPMI 1640 complemented with 1 μg/mL phorbol 12-myristate acetate (PMA) in 96-well plates (Corning, NY, USA). After overnight incubation at 37 °C, the culture media were replaced with MO suspensions in BEGM or RPMI 1640 media at concentrations ranging from 0 to 200 μg/mL, followed by 24 h incubation. Then the ATP or MTS assay solutions were added to evaluate cell viabilities by measuring the luminescence or absorbance on a SpectraMax M5 microplate spectrophotometer.

### Assessment of acute toxicological responses in the mouse lung

Male C57Bl/6 mice (8 weeks old) from Charles River Laboratories (Hollister, CA, USA) were used to test the coated and uncoated MOx. The animals were housed in a standard laboratory conditions by UCLA guidelines as well as the NIH Guide for the Care and Use of Laboratory Animals (DHEW78-23). Mice were exposed to 2 mg/kg MOx by oropharyngeal aspiration procedure as described before [10]. In detail, a total volume of 100 μL ketamine (100 mg/kg)/xylazine (10 mg/kg) was intraperitoneally injected to anesthetize the animals. The anesthetized animals were held in a vertical position to instill 50 μL PBS suspension containing 50 μg nanoparticles at the back of the tongue to allow pulmonary aspiration. Then the animals were placed to lay on their backs for recovery. The positive control group in each experiment received 5 mg/kg crystalline silica in the form of quartz particles (Min-U-Sil). After 40 h exposure, animals were sacrificed to collect bronchoalveolar lavage fluid (BALF) and lung tissues. IL-6, MCP-1 and LIX productions were measured in the BALF by ELISA. LDH level in BALF was determined by CytoTox 96® Non-Radioactive Cytotoxicity Assay kit form Promega (Madison, WI, USA) Lung tissue was fixed and stained with haematoxylin and eosin (H&E) to examine inflammations.

### Statistical Methods

Mean and standard deviation (SD) were calculated for each parameter. Results were expressed as mean SD of multiple determinations. Comparisons between groups were evaluated by two-side Student's t-test or one-way ANOVA. A statistically significant difference was assumed to exist when p was <0.05.

## Results

### Characterizing the surface reactivity of MOx and determining their cytotoxicity

**Fig. 1:**
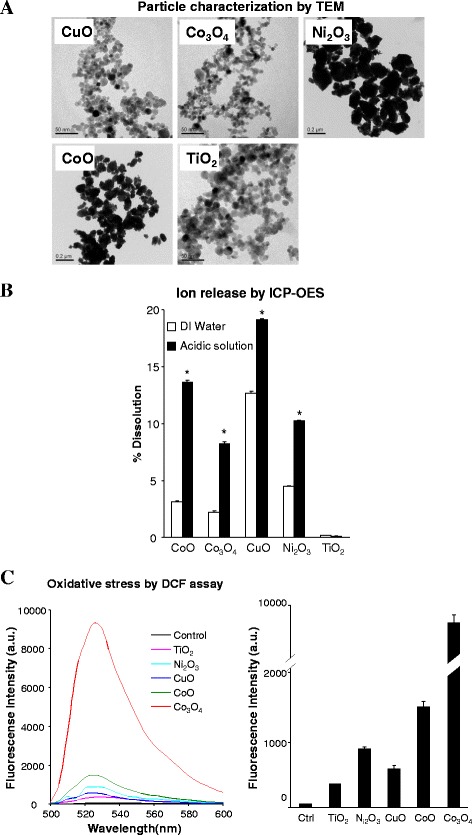
Characterizing the physicochemical properties of MO nanoparticles. **a** TEM images showing the shape and size of MOx, **b** Dissolution percentage of MO NPs in DI water and acidic solution, and **c** Determining the oxidative capability of MOx by DCF assay. The dissolution analysis was performed by suspending 50 μg/mL of each of the nanoparticles in deionized water or acidic solution (pH 4.5, HCl), followed by incubation at room temperature for 24 h. The supernatants were collected by centrifugation at 20000 g for 30 min and digested for ICP-OES measurement. For DCF assay, 200 μg/mL MOx suspensions were incubated with DCF solutions at 25 μg/mL for 2 h. The fluorescence emission spectra of the mixed solutions were collected at 500–600 nm with excitation at 490 nm. * *p* < 0.05 compared to the ion release in water

First, we established a library of MOx including CoO, Ni_2_O_3_, Co_3_O_4_, CuO, which have been shown to induce cell death by oxidative stress mechanism, and TiO_2_ was used as negative control. The shape, size and surface charge of these nanoparticles were characterized by TEM, DLS and zeta-potential analysis. As shown in Fig. 1a, primary particle sizes, as determined by TEM, are in the range of 10–100 nm. The hydrodynamic sizes assessed by DLS in water, as well as BEGM and RPMI tissue culture media, show a range from 200 to 600 nm, indicating that MOx tend to agglomerate in media (Additional file 1: Table S1). These particles show very similar zeta potential at 5 to −10 mV in cell culture media, which reflects the formation of protein corona on particle surface.

Since the mechanism of MO-induced toxicity may involve oxidative stress that can be linked to surface reactivity (*e.g*., band gap) or dissolution, we determined the dissolution rate and ability to generate ROS by MOx. Because MOx are mainly internalized into acidic lysosomes in cells (Additional file 2: Figure S1), we studied the dissolution rate of MOx in deionized water and acidic solution (HCl, pH 4.5), which has same pH but less component-complexity to artificial lysosomal fluids [25]. The released metal ions were detected by ICP-OES. As shown in Fig. 1b, compared to the dissolution rate in deionized water, CoO, Co_3_O_4_, CuO and Ni_2_O_3_ have significantly increased metal ion release at pH 4.5, while TiO_2_ is very stable in both deionized water and acidic solutions. CuO is the most dissoluble particle (19.11%) in acidic solution, while the dissolution of Co_3_O_4_ (8.23%) is relatively low compared to CuO, Ni_2_O_3_ (10.23%) and CoO (13.65%).

We used DCF assay to investigate the oxidative potential of MOx. The DCF assay is based on a mechanism that nonfluorescent H_2_DCFDA could be converted to the highly fluorescent DCF by oxidation. This assay has been widely used to access the abiotic ROS generation on nanoparticle surface [15]. As shown in Fig. 1c, while TiO_2_ and CuO show very limited oxidative potential, Ni_2_O_3_ and CoO induce substantial increases in ROS generation and Co_3_O_4_ exhibits the highest level.

**Fig. 2:**
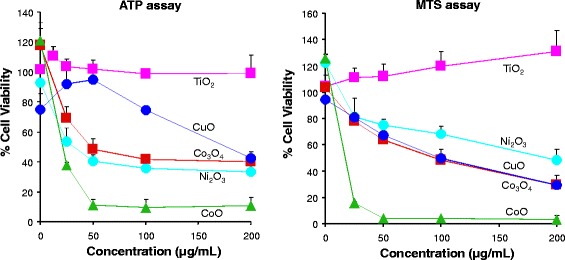
Assessment of Cell viability in BEAS-2B cells exposed to MOx. Cell viability tests were performed by ATP (*left panel*) MTS (*right panel*) assays. BEAS-2B cells were exposed to MOx suspensions (0–200 μg/mL) for 24 h. Then the MTS or ATP assay solutions were added to evaluate cell viabilities by measuring the absorbance or luminescence on a SpectraMax M5 microplate spectrophotometer

Shedding of toxic ions or ROS generation could lead to cytotoxicity. We examined cell viability in THP-1 and BEAS-2B cells by MTS and ATP assays to measure the cellular metabolic activity. The results showed that CoO, Ni_2_O_3_, Co_3_O_4_ and CuO induced significant cell death in THP-1 and BEAS-2B cells, while TiO_2_ has no effect in cell viability (Fig. 2 and Additional file 3: Figure S2). These results are consistent with previous reports that used RAW 264.7, A549 and HeLa cells [26–28]. The cytotoxicity of MOx has good correlation with the surface activity or dissolution of MOx, which are the key physicochemical properties that induce cell death.

### Engineering safe coating approach for CuO nanoparticles

In order to lower the cytotoxicity induced by MOx, we screened a series of coating agents. Herein, we compared the cytotoxicity of uncoated CuO and coated CuO by citrate, PVP and EDTMP to evaluate the effect of these coating agents. As shown in Additional file 4: Figure S3, citrate shows very limited protective effect, PVP has a moderate effect, while EDTMP is the most effective.

**Fig. 3:**
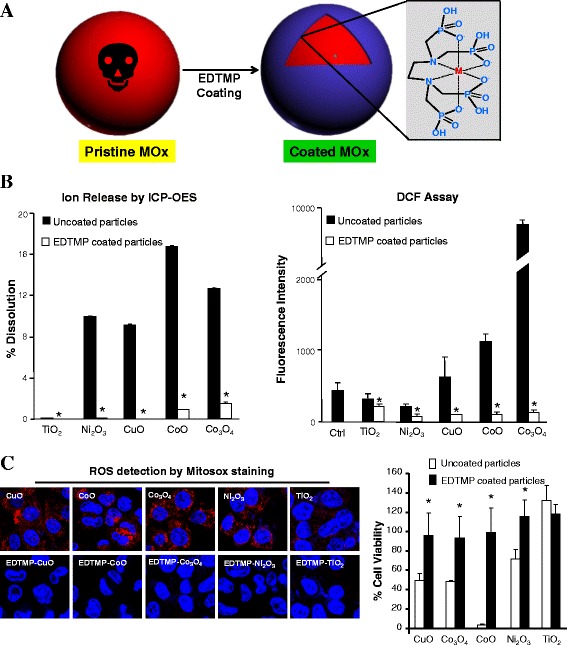
Safe design of MOx by surface passivation. **a** Formation of EDTMP-metal conjugate on MOx surface, **b** Ion release and oxidative capability of uncoated and EDTMP coated MOx, and **c** Comparison of ROS generation and cell viabilities in BEAS-2B cells exposed to uncoated and EDTMP coated MOx. The measurement of ion release and oxidative capability was described in the legend of Fig. 1. After 16 h exposure of BEAS-2B cells to 100 μg/mL MO suspensions, intracellular ROS generation was visualized by Mitosox staining. The cell viabilities were determined by MTS assay in BEAS-2B cells after 24 h incubation with 200 μg/mL MO suspensions. * *p* < 0.05 compared to uncoated MOx

We characterize the EDTMP coated CuO (EDTMP-CuO) by FTIR, TEM and XRD to understand the interaction between EDTMP molecules and CuO NPs. As shown in Additional file 5: Figure S4A, we compared the spectra of EDTMP, EDTMP-CuO and CuO. CuO particles show signature peaks of Cu-O stretching at 529 and 591 cm^−1^ bands [29]. The absorption peaks at 1626 cm^−1^ and 3443 cm^−1^ bands could be attributed to the bending vibration of hydroxyl groups or some carbonate on the surface of CuO [29]. After EDTMP coating, the intensities of -OH and carbonate peaks have a significant decrease. The additional peaks at 1109 cm^−1^ and 1048 cm^−1^ from EDTMP-CuO could be attributed to the symmetric and antisymmetric phosphonate stretching vibrations of EDTMP [30]. The XRD analysis shows that EDTMP coating does not change the crystal structure of CuO (Additional file 5: Figure S4B). Both coated and uncoated particles have similar morphology (Additional file 5: Figure S4C). The schematic in Fig. 3a shows the predicted structure of the EDTMP-Cu conjugate. The organophosphate ligand coordinates to the metal atoms in a hexadentate fashion, involving two nitrogen and four oxygen atoms. The tetrahedral phosphonic groups in this complex limit the free space around the central metal atoms, which are shielded from interacting with ligands in biological environment such as protons, biomolecules, *etc*. Since EDTMP was reported to have strong binding affinity with heavy metal ions [30–32], this coating may be used for a broad spectrum of toxic MOx to provide stable inert surfaces.

### Extending EDTMP coating to other toxic MOx

We further applied this coating to other MOx including TiO_2_, Ni_2_O_3_, Co_3_O_4_ and CoO, and demonstrated that EDTMP coating could effectively reduce the ion shedding and oxidative stress on the surface of toxic MOx. As shown in Fig. 3b, after coating, there is less than 2% ion release for Co_3_O_4_ and CoO nanoparticles in acidic solution, while Ni_2_O_3_ and CuO particles only show <0.2% dissolution. The ion shedding from toxic MOx reduced 85-99% after EDTMP coating. The DCF result shows that the oxidation potentials of these MOx decrease 65-98%, and almost get to background level after coating. Besides, we used Mitosox staining to visualize ROS production (red dots) in cells treated with MOx. Compared to the generation of massive red dots by CuO, Ni_2_O_3_, Co_3_O_4_ and CoO particles, very limited red dots could be observed in cells treated with EDTMP coated MOx (Fig. 3c). The cytotoxicity results also show that EDTMP coating could completely eliminate the cytotoxicity of MOx in BEAS-2B and THP-1 cells (Fig. 3c and Additional file 6: Figure S5). In addition, we found EDTMP coating does not interfere in the cellular uptake mechanism (endocytosis) of MOx (Additional file 7: Figure S6). Although EDTMP coating shows a little interference on the cellular uptake levels of some MOx (Additional file 8: Figure S7), these inferences cannot explain the protective effects of EDTMP coating layer. These results suggested that the surface passivation rather than cellular uptake interference by EDTMP coating is responsible for the reduced cytotoxicity of all coated MOx, and EDTMP coating could be potentially used as a safe design approach for a broad-spectrum of MOx.

### Validation of the protective effect of EDTMP coating in animals

**Fig. 4:**
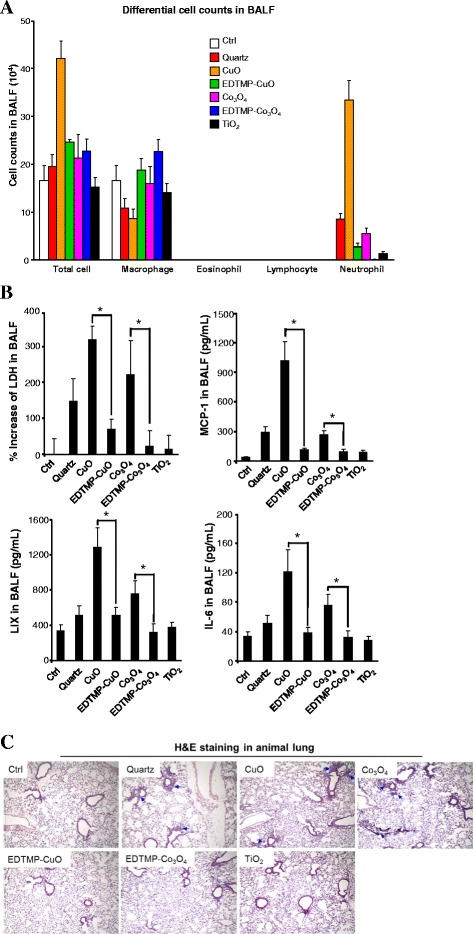
Safety assessment of EDTMP coated MOx in animal lungs. **a** Differential cell counts in BALF, **b** LDH and cytokine release in BALF, **c** H&E staining of lung sections from MO-exposed mice. TiO_2_, CuO, EDTMP-CuO, Co_3_O_4_ or EDTMP-Co_3_O_4_ were oropharyngeally administrated at 2 mg/kg (6 mice in each group), while animals received 5 mg/kg quartz exposure were used as positive control. After 40 h, animals were sacrificed to analyze LDH (*upper left panel*), MCP-1 (*upper right panel*), LIX (*lower left panel*) and IL-6 (*lower right panel*) production in BALF. * *p* < 0.05 compared to uncoated MOx

In order to further validate EDTMP coating in animals, mice were exposed to Quartz (positive Ctrl), CuO, Co_3_O_4_, EDTMP-CuO, EDTMP-Co_3_O_4_ and TiO_2_ nanoparticles by oropharyngeal aspiration because inhalation is one of the most common exposure route of MOx to humans. After 40 h exposure, the animals were sacrificed to collect BALF and lung tissues. The LDH and cytokine release in BALF were determined by LDH assay and ELISA, respectively. As shown in Fig. 4a, most of the inflammatory cells induced by uncoated MOx are neutrophils. CuO and Co_3_O_4_ induce significant release of LDH in BALF (Fig. 4b), indicating substantial tissue damage induced by MOx. In addition, both CuO and Co_3_O_4_ result in increased production of cytokines or chemokines including IL-6, MCP-1 and LIX. Compared with uncoated particles, EDTMP coating could significantly reduce the production of LDH, IL-6, MCP-1 and LIX. H&E staining also shows that CuO and Co_3_O_4_ can induce severe inflammation in animal lungs, while EDTMP coated particles have no inflammatory effects (Fig. 4c). Pristine TiO_2_, as a negative control, induces very limited hazard effects in animal lungs. All these animal results demonstrated that EDTMP coating could be used as an effective safe design approach for MOx *in vivo*.

## Discussion

This study showed that surface reactivity including ion shedding and oxidative capacity is the key physicochemical property that is responsible for the toxicity of MOx. In order to reduce or eliminate the toxicity of MOx, we examined a series of coating molecules to passivate the surface of MOx and successfully identified EDTMP, which could provide the most inert surface for toxic MOx including CuO, Co_3_O_4_, Ni_2_O_3_ and CoO. EDTMP coated MOx showed limited toxicity in THP-1 and BEAS-2B cells. Moreover, this coating could significantly reduce the production of neutrophil counts, LDH, MCP-1, LIX and IL-6 in BALF as well as inflammation responses in animal lungs exposed to MOx for 40 h.

To justify the relevance of our toxicity test to pulmonary damage in humans, we have to consider real life exposure scenarios in the workplace. Although we were not able to include human exposures in this study, recent studies have shown that when the lung burden and state of nanoparticle dispersion are kept similar, short-term inhalation exposures in rodents could be evaluated by the pulmonary responses to bolus exposure (such as oropharyngeal instillation) [33, 34]. In order to extrapolate the doses used in our exposures to MOx exposure in human, we used reported data showing that the mean values of eight-hour average particulate concentrations measured during welding at the welders’ breathing zone (0.5 m) could be as high as 11.3 mg/m^3^ [34]. In addition, NIOSH has proposed recommended limits of exposure as 1 mg/m^3^ for CuO fume, 2.4 mg/m^3^ for fine TiO_2_. Based on the calculation approach for nanomaterials being used at NIOSH [35–37], we could estimate that a worker exposure of 1–11.3 mg/m^3^ MOx for 8 h/day over a five month time period over a 10-year working time could lead to a lung burden similar to a bolus exposure of 1–12 mg/kg in the mouse. This dose range is equivalent to 0.5-6 mg/m^2^ of MOx exposure in the mouse lung, assuming the alveolar epithelium surface area of 0.05 m^2^ and a body weight of 25 g [38]. Assuming that the *in vitro* MOx dose is homogeneously distributed in cell culture media (100 μL/well, 96 well plate) and the percentages of MOx settlement are higher than 70% [39], the *in vitro* cell exposure dose would be 1.6-16 μg/mL. The *in vivo* dose used in this study is 2 mg/kg, which has previously been shown to be on the steep part of the dose response curve for MOx and relevant to the airborne levels of MOx in workplace [13]. The doses used in cell experiments range from 0 to 200 μg/mL, which is also comparable to the extrapolated *in vitro* doses (1.6-16 μg/mL). These estimates suggest that the MOx doses tested in cells and mice in this study are relevant to occupational exposures in humans. However, this *in vitro* dosage is only an estimation based on several assumptions, and more sensitive measurements and exposure models for MOx may be needed to better estimate the *in vitro* exposure dose [40].

MOx have been reported to elicit a series of adverse outcome pathways (AOPs) including ROS generation [5], mitochondrial damage [6], cytochrome C release [7], DNA damage [8], inflammatory cytokine release [9–11], disruption of autophagic flux [11, 41], *etc*. Although the AOPs are largely influenced by cell types and their states of differentiation, the nano-bio interactions of MOx have been considered to play a major role. The physical, chemical or electronic transaction would take place between the surfaces of MOx and the surfaces of biological components at the nano-bio interface. Zhang et al. have proved that the band gap and Fermi energy level of MOx determine the overlap of E_c_ levels of particles and the biological redox potential [13, 15]. This allows electron transfer from the biological redox couples to the surface of MOx, causing disruption of cellular redox homeostasis and induction of oxidative stress. Our previous studies have showed that ion shedding from the surfaces of MOx could also lead to severe hazard effect by mitochondria-mediated apoptosis or stripping of phosphate groups from biomolecules [10, 42]. Thus, surface reactivity is the dominated physicochemical property of MOx, resulting in adverse biological outcomes.

The study of the relationship between the surface reactivity of MOx and their biological effects provides opportunities for safe design of MOx. Although some approaches including coating [16, 17], core-shell structure [18, 19], doping [20] and geometric control [21] have been used to reduce the hazard effects of MOx, there is lacking of a simple method for a broad spectrum of toxic MOx. In this study, we chose surface coating, and EDTMP was identified as the most effective coating agent to passivate MOx surfaces. MOx particles have high binding affinity with EDTMP because of the unique complexation of metal atoms on particle surfaces. The metal atoms could stably chelate with the organophosphate ligand in a hexadentate fashion, involving two nitrogen and four oxygen atoms. The interaction between the metal atoms on the surface of MOx and the biological components could be significantly shielded because the tetrahedral phosphonic groups of EDTMP limit the free space around the metal atoms. As a result, the EDTMP coating is very stable, even under acidic biological conditions. Since EDTMP has been reported as an effective chelate for a variety of heavy metal ions, including Co, Zn, Cu, Mn, *etc*. [30–32], EDTMP coating could be used as an effective safe design approach for a broad spectrum of high toxic MOx.

Beside of the reduction of hazard effects, a valuable safe-by-design strategy must not significantly interfere with the potential application value of the nanomaterial. Because of their advanced properties, nanomaterials have been applied in many fields. Some of their utilities (e.g. pigment, cosmetics, etc.) may lead to high exposure risk and threaten human health, while MOx in some of their applications (e.g. mechanical parts, electronic products, etc.) may have limited exposure to humans. Although it’s hard to find a universal safe-by-design strategy that has no interferences for all of their potential application values, it’s possible to explore a safe design method that can reduce the hazard effects of nanomaterials during their applications with high exposure risk. In terms of the wide applications of MOx in pigments [43, 44], our EDTMP coating has negligible interferences in the visible light absorbance of MOx (380–780 nm), suggesting that our safe-by-design approach does not affect the applications of MOx in pigments.

## Conclusions

In this communication, we developed an effective safe design method for toxic MOx through surface coating. We selected CuO, Ni_2_O_3_, CoO and Co_3_O_4_ for the study, and they could induce toxicity by ion shedding or ROS generation on their surface. Among the coating agents, we successfully identified EDTMP to be the most effective in reducing ROS generation and dissolution, resulting in decreased cytotoxicity. This protection effect of EDTMP to MOx has been further validated in animal lungs. This EDTMP-based safe design approach has the potential to be applied to a broad-spectrum of MOx based nanoproducts, which will significantly reduce their hazard effects in humans, facilitate the safe and sustainable development of nanotechnology.

## Additional files

Additional file 1: Table S1. Zeta potential and hydrodynamic size of uncoated and EDTMP coated MOx. (PDF 64 kb)

Additional file 2: Figure S1. Intracellular distribution of MOx by confocal imaging. THP-1 cells were exposed to 25 μg/mL TiO_2_, Co_3_O_4_ or CuO nanoparticle suspensions for 16 h. Then the cells were stained with Hoechst 33342 and Alexa Fluor 594 labeled anti-LAMP1 to visualize the nuclei and lysosomes, respectively, by SP2 1P/FCS and Leica confocal SP2 MP-FLIM microscope. (PDF 158 kb)

Additional file 3: Figure S2. Assessment of cell viability in THP-1 cells exposed to MOx. THP-1 cells were exposed to MOx suspensions for 24 h. The cell viability was tested by MTS (left panel) or ATP (right panel) assay by measuring the absorbance or luminescence on a SpectraMax M5 microplate spectrophotometer. (PDF 83 kb)

Additional file 4: Figure S3. Cell viabilities of PVP, citrate, EDTMP coated and uncoated CuO NPs. THP-1 or BEAS-2B cells were exposed to EDTMP, citrate, PVP coated and uncoated CuO NPs for 24 h. The cell viability was tested by MTS assay. (PDF 83 kb)

Additional file 5: Figure S4. Characterization of CuO particles following EDTMP coating. A) TEM images of uncoated and coated CuO particles, B) FTIR spectra of uncoated and EDTMP-coated CuO as well as EDTMP by itself and C) XRD spectra of uncoated and coated particles. Both the coated and uncoated CuO particles show similar sphere shape with size at 10-20 nm. The presence of an EDTMP coating is demonstrated by characteristic P=O peaks. XRD analysis indicates that EDTMP coating do not change the crystal structure of CuO NPs. (PDF 272 kb)

Additional file 6: Figure S5. Viabilities of THP-1 cells exposed to uncoated and EDTMP coated MOx. After 24 h exposure of THP-1 cells 200 μg/mL MO suspensions, the cell viabilities were determined by MTS assay. (PDF 98 kb)

Additional file 7: Figure S6. Influence of EDTMP coating to the cellular uptake mechanism of MOx. BEAS-2B cells pretreated with 5 μg/mL cytochalasin D for 3 h were incubated with 50 μg/mL nanoparticles for an additional 6 h. The cellular uptake levels of MOx were determined by ICP-OES. (PDF 124 kb)

Additional file 8: Figure S7. Cellular uptake levels of coated and uncoated MOx. THP-1 or BEAS-2B cells were treated with 50 μg/mL nanoparticles for 6h. After thorough washing, the cells were lysed to determine the protein concentrations as well as the metal elements. * *p* < 0.05 compared to uncoated particles. (PDF 124 kb)

## Abbreviations

AOPs: Adverse outcome pathways
BALF: bronchoalveolar lavage fluid
DCF: 2',7'-dichlorofluorescein
DLS: Dynamic light scattering
EDTMP: Ethylenediamine tetra (methylene phosphonic acid)
FTIR: Fourier transform infrared
H&E: Haematoxylin/eosin
H_2_DCFDA: 2’,7’-dichlorodihydro-fluorescein diacetate
ICP-OES: inductively coupled plasma optical emission spectrometry
LDH: lactate dehydrogenase
MOx: metal oxides
NPs: nanoparticles
PMA: phorbol 12-myristate acetate
PVP: poly(vinylpyrrolidone)
ROS: reactive oxygen species
SD: standard deviation
TEM: Transmission electron microscopy
