# Erratum to: Reduction of pulmonary toxicity of metal oxide nanoparticles by phosphonate-based surface passivation

**DOI:** 10.1186/s12989-017-0216-2

**Published:** 2017-08-29

**Authors:** Xiaoming Cai, Anson Lee, Zhaoxia Ji, Cynthia Huang, Chong Hyun Chang, Xiang Wang, Yu-Pei Liao, Tian Xia, Ruibin Li

**Affiliations:** 1 Center for Genetic Epidemiology and Genomics, School of Public Health, Jiangsu Key Laboratory of Preventive and Translational Medicine for Geriatric Diseases, Medical College of Soochow University, Suzhou, 215123 China; 2 School for Radiological and Interdisciplinary Sciences (RAD-X), Collaborative Innovation Center of Radiation Medicine of Jiangsu Higher Education Institutions, Jiangsu Provincial Key Laboratory of Radiation Medicine and Protection, Soochow University, Suzhou, 215123 China; 3 Department of Medicine, University of California, Los Angeles, CA 90095 USA; 4 California NanoSystems Institute, University of California, Los Angeles, CA 90095 USA; 5 Department of Chemical and Biomolecular Engineering, University of California, Los Angeles, CA 90095 USA; 6 Department of Microbiology, Immunology, and Molecular Genetics, University of California, Los Angeles, CA 90095 USA

## Erratum

After the publication of this work [1] it was noticed that Acknowledgement statement was incorrect. The original statement reads: This work was supported by the grant from the National Natural Science Foundation of China (No. 31671032; 31,570,899), and a project funded by the Priority Academic Program Development of Jiangsu Higher Education Institutions (PAPD). R.L. is supported by the recruitment program of Global Youth Experts of China. However the correct Acknowledgement statement should read as:
