# Supplementary material for: Reduction of pulmonary toxicity of metal oxide nanoparticles by phosphonate-based surface passivation
Source: Part Fibre Toxicol. 2017 Apr 21;14:13. doi: 10.1186/s12989-017-0193-5 (PMC5399805; doi:10.1186/s12989-017-0193-5)
Supplement: Supplementary file 1 — Zeta potential and hydrodynamic size of uncoated and EDTMP coated MOx. (PDF 64 kb) [file 12989_2017_193_MOESM1_ESM.pdf]

Table S1 Zeta potential and hydrodynamic size of uncoated and EDTMP coated MOx

Nanoparticles		Zeta Potential (mV)			Hydrodynamic size (nm)		
		H_2O	RPMI	BEGM	H_2O	RPMI	BEGM
Ni_2O_3	No Coating	-24.70±1.86	-5.33±1.17	-7.31±1.28	523.7±1.6	641.9±24.4	389.7±3.1
	EDTMP	-55.08±2.82	-3.94±0.17	-1.84±5.89	274.2±3.4	545.2±5.7	499.6±6.0
TiO_2	No Coating	-7.85±0.51	11.21±4.29	7.95±4.27	435.1±3.3	608.8±8.7	515.2±10.2
	EDTMP	-35.29±1.06	-2.60±4.74	-3.97±7.22	469.1±13.2	545.2±5.7	551.8±13.0
CuO	No Coating	23.26±0.40	-0.59±13.12	-2.19±4.41	270.1±5.5	323.2±7.5	457.5±5.5
	EDTMP	-29.01±1.57	-10.39±2.56	-7.65±2.50	198.2±7.5	203.1±5.6	561.9±22.8
CoO	No Coating	29.40±0.39	-10.27±3.85	-0.66±6.36	369.1±22.7	351.8±8.4	403.7±4.3
	EDTMP	-22.81±1.61	-9.15±4.77	-6.18±2.26	252.7±11.7	232.5±1.9	326.1±7.1
Co_3O_4	No Coating	21.00±2.36	4.56±6.36	-2.81±6.34	221.3±2.9	444.9±2.5	663.5±21.9
	EDTMP	-23.09±0.90	2.01±3.79	-1.60±11.50	184.8±2.2	338.1±16.3	447.3±3.9
