# Supplementary material for: Reduction of pulmonary toxicity of metal oxide nanoparticles by phosphonate-based surface passivation
Source: Part Fibre Toxicol. 2017 Apr 21;14:13. doi: 10.1186/s12989-017-0193-5 (PMC5399805; doi:10.1186/s12989-017-0193-5)
Supplement: Supplementary file 2 — Intracellular distribution of MOx by confocal imaging. THP-1 cells were exposed to 25 μg/mL TiO2, Co3O4 or CuO nanoparticle suspensions for 16 h. Then the cells were stained with Hoechst 33342 and Alexa Fluor 594 labeled anti-LAMP1 to visualize the nuclei and lysosomes, respectively, by SP2 1P/FCS and Leica confocal SP2 MP-FLIM microscope. (PDF 158 kb) [file 12989_2017_193_MOESM2_ESM.pdf]

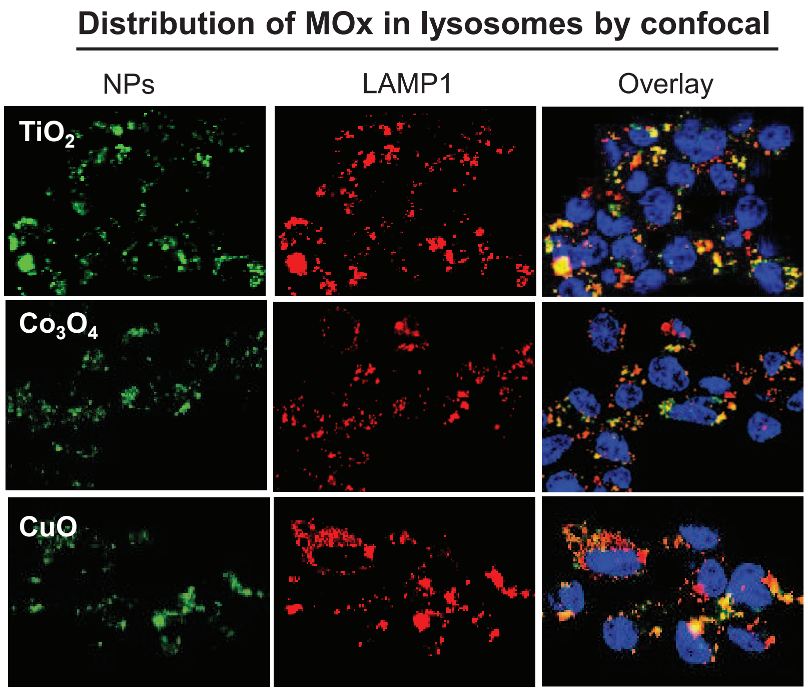

Figure S1. **Intracellular distribution of MOx by confocal imaging.**

THP-1 cells were exposed to 25 µg/mL TiO_2, Co_3O_4 or CuO nanoparticle suspensions for 16 h. Then the cells were stained with Hoechst 33342 and Alexa Fluor 594 labeled anti-LAMP1 to visualize the nuclei and lysosomes, respectively, by SP2 1P/FCS and Leica confocal SP2 MP-FLIM microscope.
