# Supplementary material for: Reduction of pulmonary toxicity of metal oxide nanoparticles by phosphonate-based surface passivation
Source: Part Fibre Toxicol. 2017 Apr 21;14:13. doi: 10.1186/s12989-017-0193-5 (PMC5399805; doi:10.1186/s12989-017-0193-5)
Supplement: Supplementary file 3 — Assessment of cell viability in THP-1 cells exposed to MOx. THP-1 cells were exposed to MOx suspensions for 24 h. The cell viability was tested by MTS (left panel) or ATP (right panel) assay by measuring the absorbance or luminescence on a SpectraMax M5 microplate spectrophotometer. (PDF 83 kb) [file 12989_2017_193_MOESM3_ESM.pdf]

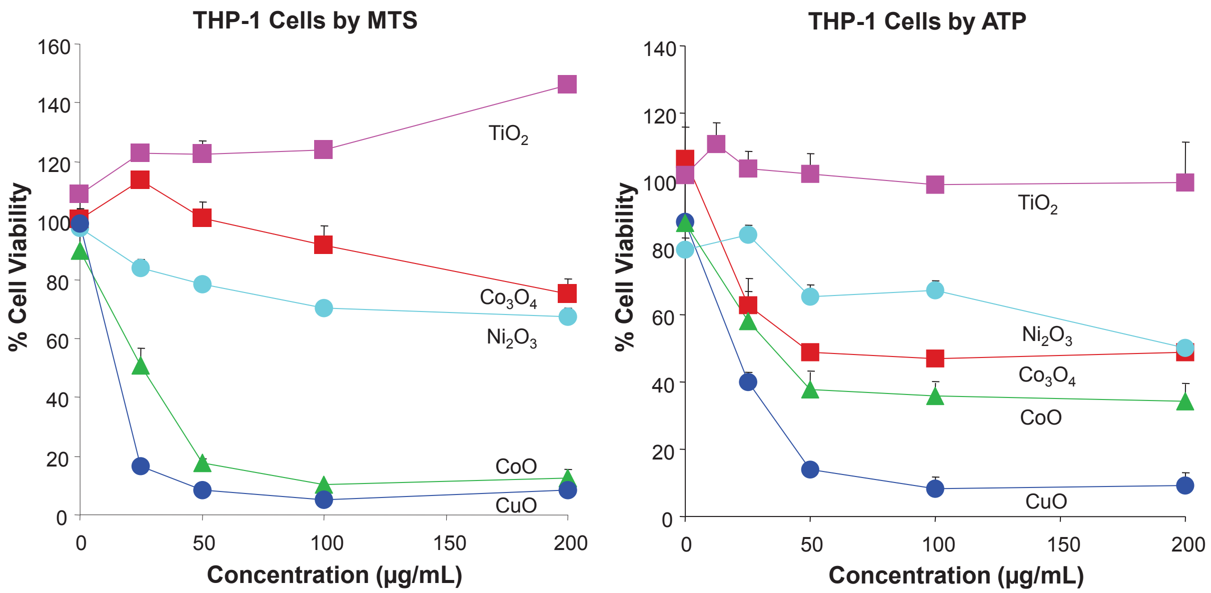

Figure S2. **Assessment of cell viability in THP-1 cells exposed to MOx.**

THP-1 cells were exposed to MOx suspensions for 24 h. The cell viability was tested by MTS (left panel) or ATP (right panel) assay by measuring the absorbance or luminescence on a SpectraMax M5 microplate spectrophotometer.
