# Supplementary material for: Reduction of pulmonary toxicity of metal oxide nanoparticles by phosphonate-based surface passivation
Source: Part Fibre Toxicol. 2017 Apr 21;14:13. doi: 10.1186/s12989-017-0193-5 (PMC5399805; doi:10.1186/s12989-017-0193-5)
Supplement: Supplementary file 4 — Cell viabilities of PVP, citrate, EDTMP coated and uncoated CuO NPs. THP-1 or BEAS-2B cells were exposed to EDTMP, citrate, PVP coated and uncoated CuO NPs for 24 h. The cell viability was tested by MTS assay. (PDF 83 kb) [file 12989_2017_193_MOESM4_ESM.pdf]

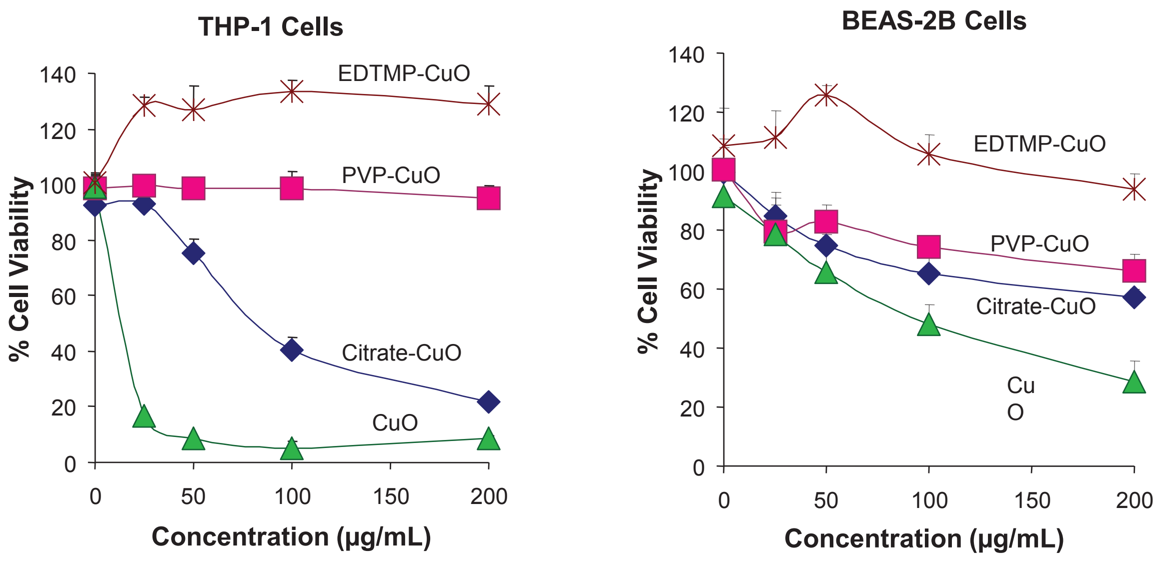

Figure S3. **Cell viabilities of PVP, citrate, EDTMP coated and uncoated CuO NPs.**

THP-1 or BEAS-2B cells were exposed to EDTMP, citrate, PVP coated and uncoated CuO NPs for 24 h. The cell viability was tested by MTS assay.
