# Supplementary material for: Reduction of pulmonary toxicity of metal oxide nanoparticles by phosphonate-based surface passivation
Source: Part Fibre Toxicol. 2017 Apr 21;14:13. doi: 10.1186/s12989-017-0193-5 (PMC5399805; doi:10.1186/s12989-017-0193-5)
Supplement: Supplementary file 5 — Characterization of CuO particles following EDTMP coating. A) TEM images of uncoated and coated CuO particles, B) FTIR spectra of uncoated and EDTMP-coated CuO as well as EDTMP by itself and C) XRD spectra of uncoated and coated particles. Both the coated and uncoated CuO particles show similar sphere shape with size at 10-20 nm. The presence of an EDTMP coating is demonstrated by characteristic P=O peaks. XRD analysis indicates that EDTMP coating do not change the crystal structure of CuO NPs. (PDF 272 kb) [file 12989_2017_193_MOESM5_ESM.pdf]

A

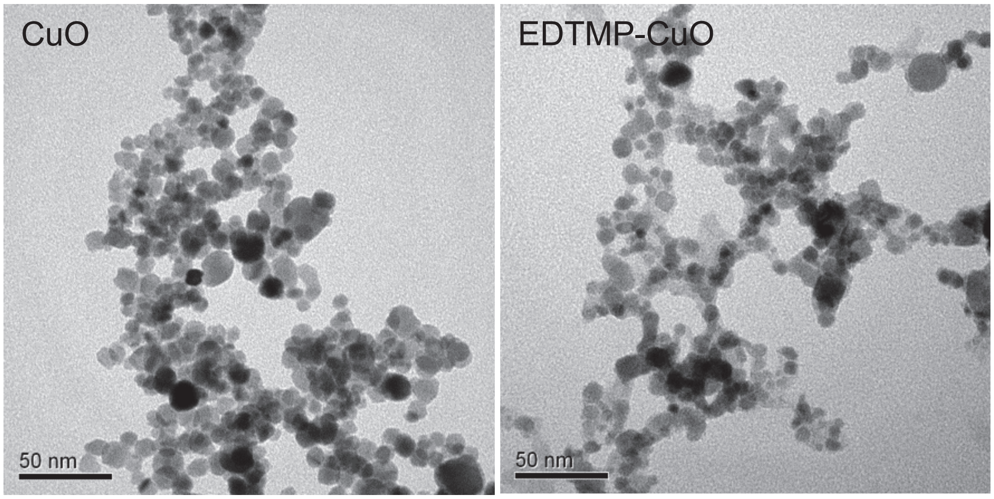
CuO
50 nm
EDTMP-CuO
50 nm

B

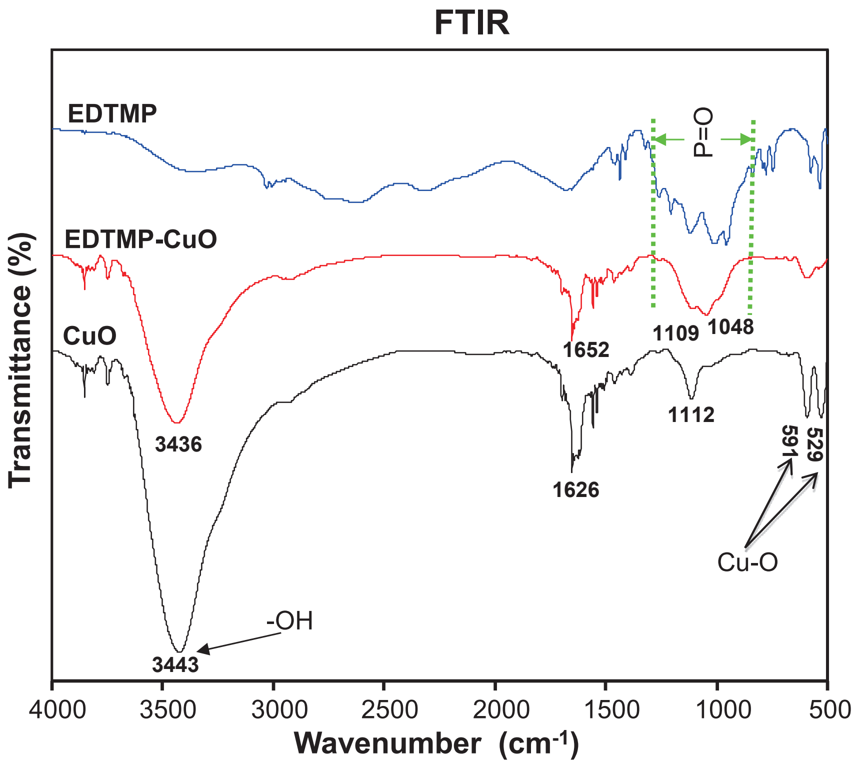
FTIR
EDTMP
P=O
EDTMP-CuO
1109
1048
1652
CuO
3436
1112
591
529
1626
Cu-O
-OH
3443
Transmittance (%)
4000
3500
3000
2500
2000
1500
1000
500
Wavenumber (cm-1)

C

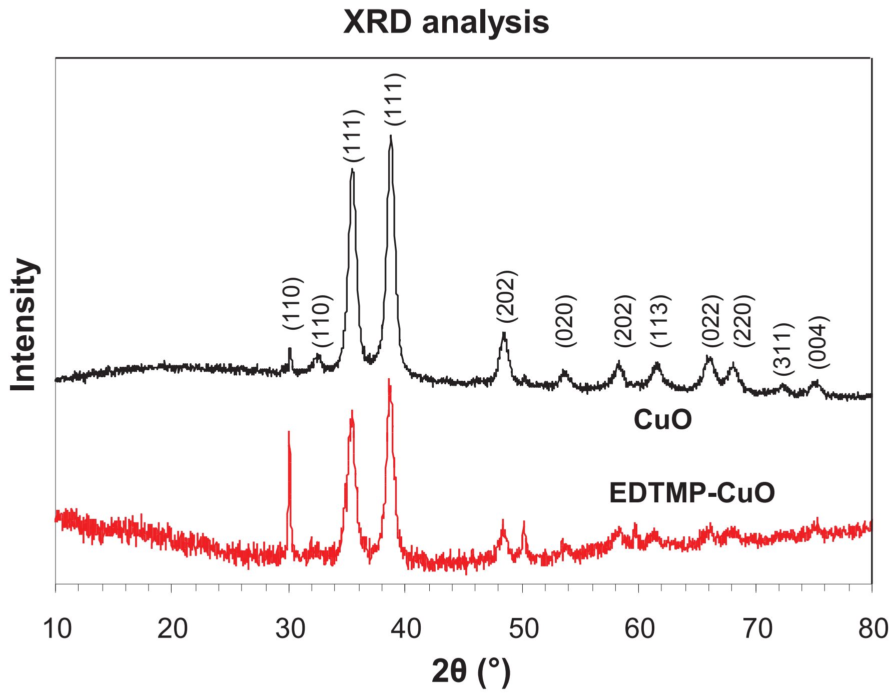

Figure S4. Characterization of CuO particles following EDTMP coating.

A) TEM images of uncoated and coated CuO particles, B) FTIR spectra of uncoated and EDTMP-coated CuO as well as EDTMP by itself and C) XRD spectra of uncoated and coated particles. Both the coated and uncoated CuO particles show similar sphere shape with size at 10-20 nm. The presence of an EDTMP coating is demonstrated by characteristic P=O peaks. XRD analysis indicates that EDTMP coating do not change the crystal structure of CuO NPs.
