# Supplementary material for: Reduction of pulmonary toxicity of metal oxide nanoparticles by phosphonate-based surface passivation
Source: Part Fibre Toxicol. 2017 Apr 21;14:13. doi: 10.1186/s12989-017-0193-5 (PMC5399805; doi:10.1186/s12989-017-0193-5)
Supplement: Supplementary file 6 — Viabilities of THP-1 cells exposed to uncoated and EDTMP coated MOx. After 24 h exposure of THP-1 cells 200 μg/mL MO suspensions, the cell viabilities were determined by MTS assay. (PDF 98 kb) [file 12989_2017_193_MOESM6_ESM.pdf]

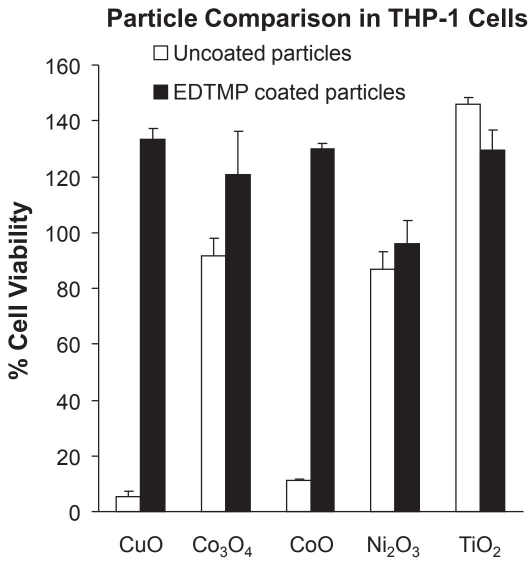

Figure S5. Viabilities of THP-1 cells exposed to uncoated and EDTMP coated MOx.

After 24 h exposure of THP-1 cells 200 μg/mL MO suspensions, the cell viabilities were determined by MTS assay.
