# Supplementary material for: Reduction of pulmonary toxicity of metal oxide nanoparticles by phosphonate-based surface passivation
Source: Part Fibre Toxicol. 2017 Apr 21;14:13. doi: 10.1186/s12989-017-0193-5 (PMC5399805; doi:10.1186/s12989-017-0193-5)
Supplement: Supplementary file 7 — Influence of EDTMP coating to the cellular uptake mechanism of MOx. BEAS-2B cells pretreated with 5 μg/mL cytochalasin D for 3 h were incubated with 50 μg/mL nanoparticles for an additional 6 h. The cellular uptake levels of MOx were determined by ICP-OES. (PDF 124 kb) [file 12989_2017_193_MOESM7_ESM.pdf]

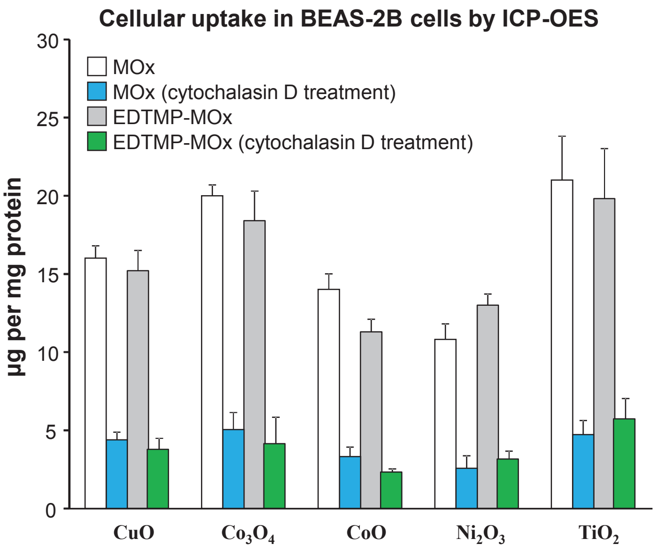

Figure S6. Influence of EDTMP coating to the cellular uptake mechanism of MOx. BEAS-2B cells pretreated with 5 µg/mL cytochalasin D for 3 h were incubated with 50 µg/mL nanoparticles for an additional 6 h. The cellular uptake levels of MOx were determined by ICP-OES.
