# Supplementary material for: Reduction of pulmonary toxicity of metal oxide nanoparticles by phosphonate-based surface passivation
Source: Part Fibre Toxicol. 2017 Apr 21;14:13. doi: 10.1186/s12989-017-0193-5 (PMC5399805; doi:10.1186/s12989-017-0193-5)
Supplement: Supplementary file 8 — Cellular uptake levels of coated and uncoated MOx. THP-1 or BEAS-2B cells were treated with 50 μg/mL nanoparticles for 6h. After thorough washing, the cells were lysed to determine the protein concentrations as well as the metal elements. * p < 0.05 compared to uncoated particles. (PDF 124 kb) [file 12989_2017_193_MOESM8_ESM.pdf]

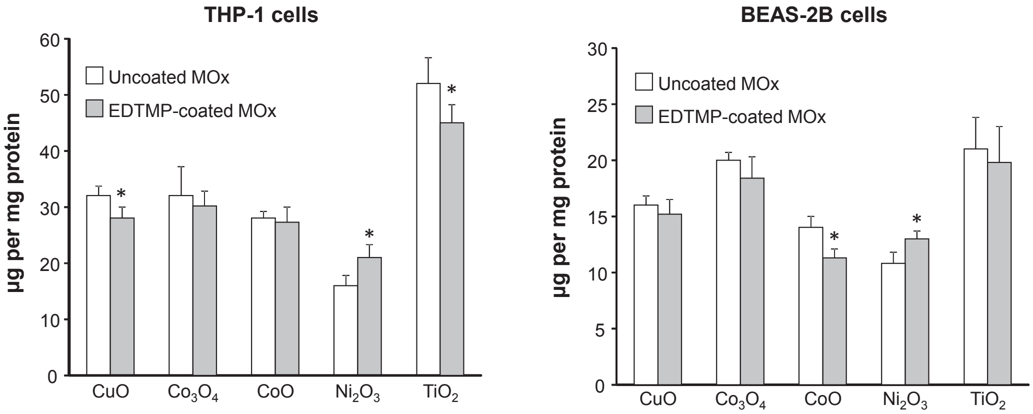

Figure S7. Cellular uptake levels of coated and uncoated MOx. THP-1 or BEAS-2B cells were treated with 50 µg/mL nanoparticles for 6h. After thorough washing, the cells were lysed to determine the protein concentrations as well as the metal elements. * $p < 0.05$ compared to uncoated particles.
